# Vaccination Perceptions of College Students: With and without Vaccination Waiver

**DOI:** 10.3389/fpubh.2018.00036

**Published:** 2018-02-21

**Authors:** Emmanuel D. Jadhav, Danielle L. Winkler, Billie S. Anderson

**Affiliations:** ^1^College of Health Professions, Ferris State University, Big Rapids, MI, United States; ^2^College of Pharmacy, Ferris State University, Big Rapids, MI, United States

**Keywords:** vaccination exempt, young adults, vaccination perceptions, campus vaccination programs, college health

## Abstract

**Introduction:**

The resurgence of vaccine preventable diseases occurs more often among intentionally unvaccinated individuals, placing at direct risk young adults not caught up on vaccinations. The objectives of this study were to characterize the sociodemographic characteristics of young adults with and without vaccination waivers and identify their perceived benefits, barriers, and influencers of vaccination.

**Methods:**

Young adults (*n* = 964) from a Midwestern rural university responded to a survey (fall 2015—spring 2016) designed to identify their perception toward vaccination. Instrument consistency was measured using the Cronbach α-scores. The Chi-square test was used to test any sociodemographic differences and Mann–Whitney *U*-tests results for differences between exempt and non-exempt students. Analysis occurred in spring 2017.

**Results:**

A little over one-third of young adults with a vaccination waiver were not up to date on their vaccinations, and think that vaccinations can cause autism. The biggest identifiable benefit was effective control against disease. The surveyed young adults ranked the out of pocket cost associated with vaccination as the most important barrier and safe and easy to use vaccines as the most important influencer of vaccination.

**Conclusion:**

Young adults who have had a vaccination waiver appear to not be up to date on their vaccinations. Vaccine administration programs, such as university campus clinics, would benefit from addressing perceptions unique to young adults with and without a vaccine waiver. This would subsequently better provide young adults a second shot for getting appropriately caught up on vaccinations.

## Introduction

In intentionally unvaccinated individuals, the incidence of vaccine preventable diseases (VPDs) is higher (1–4). The vaccination coverage in young adults for the 2016–2017 season is 33.6%, well below the national target of 70% vaccination coverage (5). University campus clinics are dealing with an increased incidence of VPDs (6–9). Despite over two centuries of vaccine availability (10, 11) and annually approved vaccination schedules for children and adults (12) the resurgence in incidence of VPDs has grown substantially (3), placing at direct risk individuals both vaccinated and non-vaccinated. Recent campus outbreaks (13), coupled with efforts (14) to highlight vaccine successes, brings to the forefront the longstanding college community concern about vaccination requirements on university campuses.

Individuals can choose to not be vaccinated by filing a vaccination exemption request. Typically, the exemption requests are submitted for young children by their parents. While there is no law on individual vaccination status, certain institutions such as public schools, and professions such as the military require an updated immunization status. Every state has well developed criteria for allowing a vaccination waiver. The criteria are broadly grounded in religious, philosophical, or medical reasons (15). In some states, such as Michigan, there has been an increase in the number of vaccination waivers among K-12 students (16). This makes the population at direct risk to contract VPDs those students who were vaccine exempt due to the signing of a vaccination waiver on their behalf as children (17–19).

As those who were under or unvaccinated as children now enter college, especially those college programs that do not require an updated vaccination status (20), they pose an increased risk of disease susceptibility (21) by living and interacting in close quarters, leading to the emergence and reemergence of serious infectious disease outbreaks (22). The empirical literature regarding the perception toward vaccination among those students who may or may not have been exempt from vaccinations as children is limited, and not much is known about their perception toward vaccinations. The objectives of this study were to characterize the sociodemographic characteristics of current university students with and without vaccination waivers as children and identify their personal perceived benefits, barriers and influencers of vaccination. Findings from this study may help university campus clinic programs to create targeted perception-based vaccine messages. Additionally, the findings could also guide the development of program practices geared toward catching-up those students who were vaccine exempt or not up to date on their vaccinations.

## Materials and Methods

### Study Sample

In this cross-sectional study that took place from fall 2015 to spring 2016, we recruited students at all campus locations of a public university in the rural Midwest of the United States. The university does not require vaccinations for students except for in certain medical programs. The survey was sent to all the university students. A survey reminder email was sent to non-respondents two weeks after the original email. The invitation emails included background information on the study and the importance of student participation, the names of the researchers, and a link to complete the survey. The email also stated that by clicking the link to complete the survey, they were providing informed consent. The survey was administered electronically by the University Administration using the QuestionPro platform and de-identified data was provided to the authors for study analysis. The study was approved by the Ferris State University Institutional Research Board.

### Instrument

The survey consisted of 15 questions. Drawing on the Health Belief Model, the instrument was designed to identify students’ perceived benefits, barriers, and influencers of vaccination (23), and measures were drawn from relevant empirical literature. Vaccination benefits were those identified by the World Health Organization and include effective control against disease, prevention of the onset of disease, protection of the unvaccinated community, savings of time and money otherwise lost to disease, and safety for those giving and receiving the vaccinations (24). The barriers included in the study were personal adverse experiences or that of a parent or guardian, the associated out of pocket costs, fear/pain of needles, lack of transportation, risk of adverse event greater than perceived benefit, and for moral or religious reasons (25, 26). To identify the cues to action (27), a construct of the Health Belief Model which identifies ways to activate willingness to engage in healthy preventive behaviors, we identified influencers such as educational material, low cost, ease of access, safe to use and administer vaccines, follow-up on compliance, and vaccination status of parent/guardian (28).

Other variables on the instrument included current vaccination status, which was the primary outcome of interest and sociodemographic characteristics such as gender, age, academic college in which enrolled, financial assistance status, current level of education, education level of parent/guardian, individual insurance status, regular access to primary care provider, and vaccine counseling and concerns related to vaccination leading to autism. We selected these sociodemographic characteristics based on available literature regarding vaccination trends (26, 28, 29). Vaccination waiver status was also measured. This question refers to having had a waiver signed for any vaccination as required for school, college, or employment.

### Statistical Analysis

Percentages for each level of the sociodemographic characteristics statistics were computed for the respondents with and without the vaccination waivers. Chi-square tests were run to test for associations between sociodemographic characteristics and the vaccination waiver status. Three questions required respondents to rate their perceived benefits, barriers, and influencers on a Likert scale of least important (rank = 1) to most important (rank = 5). Since the barriers, influencers, and benefit questions on the survey instrument were measured on a Likert scale the median values, which serves as an appropriate measure of central tendency in order to fairly characterize the responses from the instrument, are reported (30, 31). In addition, the results of the Mann–Whitney *U*-tests, which are appropriate for testing differences in median values between two groups (32), are also reported. Cronbach α on all scales that measured the benefits barriers and influencers of vaccination from the instrument are reported. All statistical tests were performed using SAS 9.4.

## Results

### Participant Characteristics

A total of 1,089 students from the university student population of 14900 students responded to the survey. Of these 1,089 respondents, we dropped 121 respondents as they had an incomplete response to the vaccination waiver status question, which was the primary outcome of interest in our study; and another 4 respondents were dropped because they were minors, resulting in a final sample of 964 respondents. Approximately 9% (*n* = 79) of the 964 participants responded “Yes” to having had a vaccination waiver. Of the respondents who had a vaccination waiver, 72% (*n* = 57) were females, of which 43% (*n* = 34) were in the age group of 18–21 years, and about 31% (*n* = 24) of them were from the College of Health Professions. Over 30% (*n* = 24) of them had a parent/guardian whose highest level of education was a Bachelor’s degree and over 72% (*n* = 57) had not experienced any change in their insurance status that could have affected their access to vaccines in the last three years. Statistically significant chi-square tests were detected for whether the respondent was up to date on vaccinations [χ^2^(2) = 75.62; *p* < 0.001], thinks that resurgence in VPDs is related to decline in vaccination rates [χ^2^(3) = 59.05; *p* < 0.001] and thinks that vaccinations can cause autism [χ^2^(3) = 60.53; *p* < 0.001]. These findings are represented in the Table 1.

**Table 1:** Participant characteristics by vaccination exemption status.

Category	Waiver status = yes (*n* = 79; 8.20%)	Waiver status = No/unsure (*n* = 885; 91.80%)
**Gender**		
Male	22 (27.85%)	266 (30.06%)
Female	57 (72.15%)	614 (69.38%)
Transgender	0 (0.00%)	4 (0.45%)
Missing	0 (0.00%)	1 (0.11%)

**Ages**		
18–21	34 (43.04%)	340 (38.42%)
22–25	15 (18.99%)	243 (27.46%)
26–29	6 (7.59%)	93 (10.51%)
30–33	6 (7.59%)	57 (6.44%)
34–37	4 (5.06%)	40 (4.52%)
38 or older	14 (17.72%)	112 (12.66%)
**Academic college**		
Arts and sciences	9 (11.39%)	153 (17.29%)
Business	13 (16.46%)	125 (14.12%)
Education and human services	14 (17.72%)	80 (9.04%)
Engineering technology	3 (3.80%)	84 (9.49%)
Health Professions	24 (30.38%)	210 (23.73%)
Kendall college of art and design	6 (7.59%)	52 (5.88%)
Michigan college of optometry	1 (1.27%)	13 (1.47%)
Pharmacy	8 (10.13%)	143 (16.16%)
University college	1 (1.27%)	20 (2.26%)
Missing	0 (0.00%)	5 (0.56%)

**Which of the following forms of financial assistance do you currently receive?**								
Governmental scholarships (TIP, etc.)	21 (26.58%)	202 (22.82%)
Ferris Scholarships	34 (43.04%)	423 (47.80%)
Other scholarships (community, church, rotary, etc.)	12 (15.19%)	132 (14.92%)
Reserve Officers’ Training Corps (ROTC)	0 (0.00%)	2 (0.23%)
Student Loans	46 (58.23%)	592 (66.89%)
Family Support	22 (27.85%)	286 (32.32%)
Not Sure	1 (1.27%)	8 (0.90%)
None of the above	12 (15.19%)	115 (12.99%)

**Current level of education**		
Undergraduate	58 (73.42%)	626 (70.73%)
Graduate/First Professional	21 (26.58%)	256 (28.93%)
Missing	0 (0.00%)	3 (0.34%)

**Highest level of education completed by a parent/guardian**		
High school	16 (20.25%)	243 (27.46%)
Associate’s degree	14 (17.72%)	204 (23.05%)
Bachelor’s degree	24 (30.38%)	256 (28.93%)
Graduate level degree (Master’s)	16 (20.25%)	126 (14.24%)
Terminal degree (Ph.D., MD., etc.)	5 (6.33%)	30 (3.39%)
Unsure/prefer not to answer	4 (5.06%)	25 (2.82%)
Missing	0 (0.00%)	1 (0.11%)

**Experience regarding access to physician and vaccine counseling**		
Had REGULAR access and was counseled by primary care provider	54 (68.35%)	597 (67.46%)
Had REGULAR access and was NOT counseled by primary care provider	7 (8.86%)	113 (12.77%)
Had LIMITED access and was counseled by primary care provider	5 (6.33%)	27 (3.05%)
Had LIMITED access and was NOT counseled by primary care provider	2 (2.53%)	48 (5.42%)
Had NO access to primary care provider but received vaccine counseling	0 (0.00%)	4 (0.45%)
Had access to primary care provider but never received vaccine counseling	2 (2.53%)	37 (4.18%)
Not sure about access to vaccine counseling	9 (11.39%)	58 (6.55%)
Missing	0 (0.00%)	1 (0.11%)

**Currently up to date on vaccinations**		***
Yes, currently up to date	51 (64.56%)	752 (84.97%)
No or unsure, but plan to be updated	5 (6.33%)	94 (10.62%)
No, and do not plan to be updated	23 (29.11%)	38 (4.29%)
Missing	0 (0.00%)	1 (0.11%)

**Changes in insurance coverage over the last 3 years affecting access to vaccines**		
Yes, I now have insurance	6 (7.59%)	87 (9.83%)
Yes, I have improved insurance	7 (8.86%)	59 (6.67%)
Yes, I no longer have insurance	1 (1.27%)	46 (5.20%)
No, there have been no changes to my insurance	57 (72.15%)	554 (62.60%)
Not sure	8 (10.13%)	134 (15.14%)
Missing	0 (0.00%)	5 (0.56%)

**Think resurgence in vaccine preventable diseases is related to decline in vaccination rates**		***
Yes, for sure	25 (31.65%)	536 (60.56%)
Yes, probably/not sure, could be related	38 (48.10%)	313 (35.37%)
Not at all related	16 (20.25%)	29 (3.28%)
Missing	0 (0.00%)	7 (0.79%)

**Think vaccinations can cause autism**		***
Yes, for sure	13 (16.46%)	21 (2.37%)
Yes, probably/not sure, could be related	36 (45.57%)	243 (27.46%)
Not at all related	30 (37.97%)	615 (69.49%)
Missing	0 (0.00%)	6 (0.68%)

Chi-Square test for Ho: there is no difference between samples by vaccination exemption status; all numbers rounded to two decimal places. *p < 0.05; **p < 0.01; ***p < 0.001.

### Distribution of Responses to the Benefits, Barriers, and Influencers of Vaccination

The distribution of responses are reported in Table 2. The biggest identifiable benefit was effective control against disease. The out of pocket cost associated with vaccination was the most reported barrier and vaccines that are safe to use and easy to administer was the most important influencer of vaccination.

**Table 2:** Distribution of responses to the benefits, barriers and influencers of vaccination.

Benefits, barriers and influencers	1 = least important	2 = somewhat important	3 = neutral	4 = somewhat important	5 = most important	Missing
	*N*	*N*	*N*	*N*	*N*	*N*
**Benefits**						
Effective control against disease	41 (4.24%)	25 (2.5%)	77 (7.99%)	172 (17.84%)	633 (65.66%)	16 (1.66%)
Prevents onset of disease	46 (4.77%)	41 (4.25%)	100 (10.37%)	185 (19.19%)	573 (59.44%)	19 (1.97%)
Protects the unvaccinated community	144 (14.94%)	84 (8.71%)	150 (15.56%)	118 (12.24%)	446 (46.27%)	22 (2.28%)
Saves time and money otherwise lost to disease	137 (14.21%)	101 (10.48%)	131 (13.59%)	117 (12.14%)	456 (47.30%)	22 (2.28%)
Safe to use and receive	61 (6.33%)	74 (7.68%)	98 (10.17%)	155 (16.08%)	557 (57.78%)	19 (1.97%)

**Barriers**						
Personal/parent/guardian adverse experiences	560 (58.09%)	82 (8.51%)	85 (8.82%)	71 (7.37%)	118 (12.24%)	48 (4.98%)
Out of pocket cost	442 (45.85%)	138 (14.32%)	128 (13.28%)	83 (8.61%)	131 (13.59%)	42 (4.36%)
Pain or fear of needles	492 (51.04%)	127 (13.17%)	122 (12.66%)	65 (6.74%)	119 (12.34%)	39 (4.05%)
Transportation and location	584 (60.58%)	111 (11.51%)	97 (10.06%)	65 (6.74%)	58 (6.02%)	49 (5.08%)
Moral or religious reasons	764 (79.25%)	45 (4.67%)	32 (3.32%)	27 (2.80%)	39 (4.05%)	57 (5.91%)
Risk of adverse event greater than benefit	517 (53.63%)	100 (10.37%)	95 (9.85%)	69 (7.16%)	130 (13.49%)	53 (5.50%)

**Influencers**						
Educational materials	105 (10.89%)	61 (6.33%)	174 (18.05%)	177 (18.36%)	420 (43.57%)	27 (2.80%)
Low cost vaccines	106 (11.00%)	81 (8.40%)	163 (16.91%)	176 (18.26%)	407 (42.22%)	31 (3.22%)
Parent/guardian vaccination status	289 (29.98%)	132 (13.69%)	170 (17.63%)	115 (11.93%)	219 (29.72%)	39 (4.05%)
Ease of access to vaccine programs and facilities	78 (8.09%)	74 (7.68%)	177 (18.36%)	211 (21.89%)	395 (40.98%)	29 (3.01%)
Safe to use and easy to administer	61 (6.33%)	53 (5.50%)	114 (11.83%)	190 (19.71%)	516 (53.53%)	30 (3.11%)
Follow-up on vaccine compliance	134 (13.90%)	136 (14.11)	222 (23.03%)	149 (15.46%)	286 (29.67%)	37 (3.84%)

### Benefits, Barriers, and Influencers by Vaccination Waiver Status

The Cronbach α on all scales that measured the barriers, influencers and benefits ranged between 0.80 and 0.85, which is over the acceptable 0.70 as suggested by Nunnally and Bernstien (33). The five benefit and influencer questions were statistically different between respondents with and without waivers. The two barrier questions that produced significant differences between respondents with and without waivers were about personal or parent or guardian adverse experience and risk of adverse event greater than benefit. The median rating from each of the sections on benefits, barriers, and influencers are represented in Table 3.

**Table 3:** Median responses to the benefits, barriers and influencers for respondents with and without vaccination waivers.

Benefits, barriers, and influencers of	Waiver status = Yes (*n* = 79)	Waiver status = No/unsure (*n* = 885)
	Median	Median
**Benefits**		
Effective control against disease	4.00	5.00***
Prevents onset of disease	4.00	5.00***
Protects the unvaccinated community	3.00	4.00***
Saves time and money otherwise lost to disease	3.00	5.00***
Safe to use and receive	3.00	5.00***

**Barriers**		
Personal/parent/guardian adverse experiences	3.00	1.00***
Out of pocket cost	2.00	2.00
Pain or fear of needles	1.00	1.00
Transportation and location	1.00	1.00
Moral or religious reasons	1.00	1.00
Risk of adverse event greater than benefit	4.00	1.00***

**Influencers**		
Educational materials	3.00	4.00***
Low cost vaccines	3.00	4.00***
Parent/guardian vaccination status	2.00	3.00*
Ease of access to vaccine programs and facilities	3.00	4.00***
Safe to use and easy to administer	4.00	5.00**
Follow-up on vaccine compliance	2.00	3.00***

Mann–Whitney U-test for Ho: there is no difference between samples by vaccination exemption status; all numbers rounded to two decimal places. *p < 0.05; **p < 0.01; ***p < 0.001; the median response ranks on the Likert scale correspond to; 1 = least important, 2 = slightly important, 3 = neutral, 4 = moderately important, 5 = most important.

## Discussion

The results suggest statistically distinct underlying differences between vaccination perceptions of students with and without vaccination waivers. Of particular attention is the result that among the students who had received a vaccination waiver, close to one-third of them were not up to date on their vaccinations and did not plan to be updated. This could be explained as a reflection of the identified barriers that include out of pocket cost associated with the catch-up schedule (34) or the fear associated with the risk of adverse experience with vaccination schedules (20). Likewise, the proportion of those that are not sure if they think the resurgence in VPDs is related to decline in vaccination rates may be related to the transfer of commonly held negative perceptions associated with vaccine effectiveness (35). Despite the retraction of the study which incorrectly suggested that vaccinations lead to autism, it continues to remain a popular idea (36). In the San Diego, CA, USA measles outbreak started by an intentionally unvaccinated young adult boy, over 75% of the children were intentionally not vaccinated and one of their top reasons was the belief that vaccinations cause autism (37). Our study finding for whether the respondents think that vaccinations can cause autism aligns with other studies (37) and affirm that it continues to be a negative health belief, as observed in our study participants, and more so amongst those students who have had a vaccination waiver. Interestingly the comparison of groups with limited access and with or without being counseled by a primary care provider suggests an opportunity for campus clinics to educate students on vaccination benefits and subsequently address the concern of our study that adults who were under or unvaccinated as children are now entering college in a communal environment. The finding that 65% of students that had a vaccination waiver reported as being up to date on their current vaccination further reinforces the power of health education but may also be a reflection of the high number of respondents from the College of Health Professions that run programs that require them to be up to date on vaccinations.

As demonstrated by their scores on the benefits of vaccination questions, respondents, with and without waivers, ranked vaccinations as an effective way to control and prevent the onset of disease as the highest ranked benefits. This finding aligns with another study among university students that found the number one priority of vaccination to be protection against diseases that are life threatening or that greatly impact the quality of life (38). This finding is important because it provides the university healthcare community with insight into the viewpoint of the reasons why a young adult population views vaccinations as beneficial. Hence, university health centers could devise vaccination campaigns that specifically focus on the benefits rated as most important in order to decrease vaccination waivers among students.

The five benefit questions were statistically different from each other when comparing respondents with and without waivers. The respondents with waivers rated vaccination benefits lower than the respondents without waivers. This finding would indicate that those respondents with the waivers perceive fewer benefits of vaccination as those with no waiver.

The barrier questions that produced significant differences between respondents with and without waivers both dealt with adverse events of vaccination. An informative comparison was the first barrier question that measured parent/guardian adverse experiences. This finding suggests that from a barrier perspective, the opinions of students tend to reflect their parental perceptions. Other studies also support this finding. For example, a random-effects model analyzed over 45,000 parent–child relationships and found a high correlation between parent and child attitudes and perceptions (39). However, intergenerational perceptions regarding vaccinations may be affected by race. A study regarding vaccination practices found that family traditions and the intergenerational transfer of health practices was an important source of vaccine confidence for whites, whereas for many African Americans, traditional family perceptions weakened vaccine confidence (40). Another study, particularly aimed at African American students at Howard University, found that intrapersonal factors were important when deciding whether the student would participate in a vaccination program (31). The study suggested that the optimal time to try to persuade a university student to obtain a vaccination is at the point of contemplation, during which many students understand the efficacy of the vaccine but find it difficult to obtain (31). The challenge for campus vaccination programs for students is to reign in negative intergenerational perceptions to vaccination and develop strategic, targeted messaging that inform, educate, and encourage students to confront the perception of adverse effects associated with vaccinations.

Vaccine safety was identified as the most important perceived cue to action or influencer amongst the students in both groups. This finding is consistent with other studies (41) among university students and may suggest persistent fear, reflecting distrust in healthcare professionals. The questioning of the safety of vaccinations has given the anti-vaccination community a forum, and with the advent of websites and blogs this adverse view of vaccinations can spread quickly in a viral manner (11).

This survey finding related to safety as the number one influencer of vaccination among those with and without waivers is another justification why not only vaccine receivers but even public health-care workers need to be properly educated on vaccination safety. A recent cross-sectional study found that there is still a major need for health-care workers to be properly educated on vaccination safety (42). This study suggests that improving public health worker education regarding safety of vaccinations may have the potential to decrease the number of vaccination waivers and subsequently increase vaccination coverage.

An informative comparison among the influencers of vaccination found a significant difference among low cost vaccines between the two groups. This finding is of particular relevance to rural university communities, like that of our study population. For example, in our rural community, approximately 21% of the residents are categorized as poor with a median income is $41,500. Being that the university is in a rural part of the country where the nearest metropolitan area is over 50 miles away, many students may rely on the student health center for all their preventative medical needs. Given this demographic, a community outreach program to assist students in understanding the out-of-pocket costs of vaccination and the coverage their insurance company provides for vaccinations could be useful. In addition, educating students about cost-free vaccination services that local health departments may offer and teaming up with local health departments to offer little to no cost vaccinations might be an effective way to lower the cost barriers for students.

### Limitations

We were able to meet the objectives of the study through the statistical procedures we used, and the large sample of current university students. The high Cronbach scores support the reliability of the study instrument (33) that assessed an array of benefits, barriers and influencers of vaccinations that were of interest to our study. Administering the survey online likely yielded more openness and full responses (43). The study was limited, however, in that, it was cross-sectional and only surveyed one university, thus results may not represent students in communities across the United States. In addition, the survey did not distinguish between under vaccinated and unvaccinated populations. This is a limitation because some individuals may choose to follow alternate vaccination schedules (17), leaving them temporarily under vaccinated. Up-to-date status on required vaccinations was not defined. This may have resulted in mixed results, for example based on whether or not an individual considered the HPV vaccination a requirement for up-to-date vaccination status. Another limitation with this question is that students may not know their vaccination status. Additionally, students were asked about changes in health insurance affecting access to vaccines. Many students are covered under their parents’ policies and are unaware of their insurance status, resulting in misclassification bias. One last potential limitation of this study is non-response bias. The response rate of approximately 7% may not accurately represent the study population and limits generalizability. However, the results serve to build the body of literature on perceptions of college students toward vaccination, especially in those with a vaccination waiver. In the future, alternate recruitment methods would be considered to obtain a better representation of the population under study. The authors are extending the current study to identify factors that could affect vaccination waiver status among students.

### Conclusion and Implications

In the midst of the deepening confusion over vaccination safety and the resurgence of VPDs (44), the results of our study evidence distinct variation in perceptions toward vaccination amongst students who have had a vaccination waiver and those who did not have a vaccination waiver. Furthermore, the identified perception of benefits, barriers, and cues for action or influencers could inform education and information needs of vaccination efforts offered by campus clinics. The results of this study would enable a targeted, appropriate, and relevant approach to facilitate the students’ ability to make an informed choice about vaccination safety, and subsequently, the uptake of vaccination services on university campuses and vaccination services offered by local health departments.

Our study suggests that making vaccinations convenient, affordable, and accessible is vital for a university community, particularly, in a rural setting. For students who are uncertain about whether to get vaccinated, proper educational materials that dispel myths and misconceptions and further the understanding of the risks and benefits of vaccination should be distributed to target these individuals. Potentially, greater availability of such information among a university community would diminish barriers and assist students in reaching an affirmative decision to participate in a future vaccination campaign.

## Ethics Statement

This study was approved by the “Ferris State University Institutional Review Board.” The study was exempt from documentation of written informed consent.

## Author Contributions

EJ is the project principal investigator. He conceived the study plan and drafted and revised the draft manuscript. DW performed primary analysis and drafted the initial manuscript along with revising the draft manuscript. BA analyzed the data, wrote the statistical analysis, and revised the draft manuscript. All authors reviewed and approved the final manuscript as submitted.

## Conflict of Interest Statement

BA serves as a consultant/trainer for SAS, Inc. The remaining authors have no conflicts of interest to declare.
